# Supplementary material for: Analysis of Functional Domains in Rho5, the Yeast Homolog of Human Rac1 GTPase, in Oxidative Stress Response
Source: Int J Mol Sci. 2019 Nov 7;20(22):5550. doi: 10.3390/ijms20225550 (PMC6888954; doi:10.3390/ijms20225550)
Supplement: Supplementary file 1 [file ijms-20-05550-s001.pdf]

Supplementary Figure S1

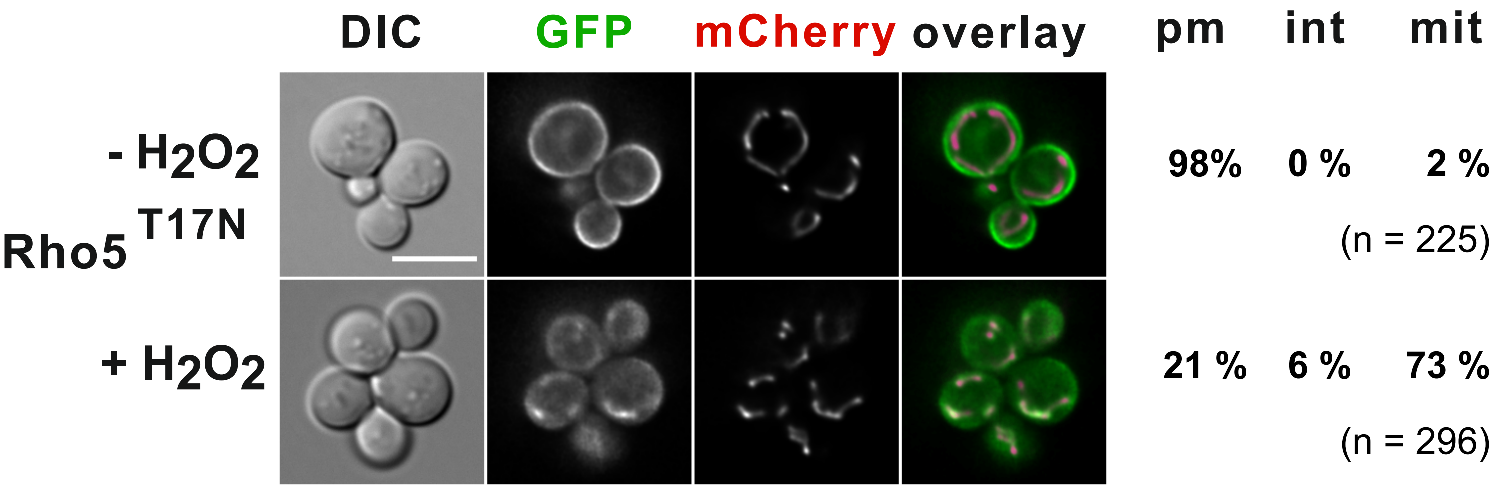

Figure S1: Life-cell fluorescence microscopy of a *rho5* deletion strain with a genomic IDP1-mCherry fusion as a mitochondrial marker (HCSO76-1A) expressing an N-terminal GFP fusion of the $Rho5^{T17N}$ variant from a *CEN/ARS* vector (pCSO92). Cells were grown on synthetic media selecting for maintenance of the plasmid and either examined without further treatment (upper panel), or after addition of 4.4 mM hydrogen peroxide for less than 15 min (lower panel). The percentage of cells displaying association of the GFP signal with the plasma membrane (pm), being cytosolic or associated with internal structures (int), and those showing colocalization with the mitochondrial marker (mit) were counted and related to the total number of cells for each sample (n) to calculate the percentage of specific localization. Images in the mCherry channel were obtained by 0.5 sec exposures, those in the GFP channel by 2 sec exposures. The size bar in the upper left DIC image represents 5 µm, applicable to all images.
